# In Silico and In Vitro Studies on an Asymmetrical Porphyrin Derivative with Therapeutic Potential in Skin Disorders

**DOI:** 10.3390/ph17060688

**Published:** 2024-05-27

**Authors:** Andreea Mihaela Burloiu, Dragos Paul Mihai, Gina Manda, Dumitru Lupuliasa, Ionela Victoria Neagoe, Radu Petre Socoteanu, Mihaela Surcel, Laurentiu-Iliuta Anghelache, Laura Olariu, Cerasela Elena Gîrd, Rica Boscencu

**Affiliations:** 1 Faculty of Pharmacy, “Carol Davila” University of Medicine and Pharmacy, 6 Traian Vuia, 020956 Bucharest, Romania; andreea-mihaela.burloiu@drd.umfcd.ro (A.M.B.); dumitru.lupuliasa@umfcd.ro (D.L.); cerasela.gird@umfcd.ro (C.E.G.); rica.boscencu@umfcd.ro (R.B.); 2 “Victor Babeş” National Institute of Pathology, 050096 Bucharest, Romania; ionela.neagoe@ivb.ro (I.V.N.); mihaela.surcel@ivb.ro (M.S.);; 3 “Ilie Murgulescu” Institute of Physical Chemistry, Romanian Academy, 060021 Bucharest, Romania; 4 SC. Biotehnos SA, 3-5 Gorunului, 075100 Bucharest, Romania; lolariu@biotehnos.com

**Keywords:** asymmetrical porphyrin, photodynamic therapy, HaCaT keratinocytes, Hs27 skin fibroblasts, carbonic anhydrase IX, molecular dynamics

## Abstract

For developing novel photosensitizers with therapeutic potential in non-malignant and malignant cutaneous disorders, the unsymmetrical porphyrin, 5-(2-hydroxy-3-methoxyphenyl)-10, 15, 20-*tris*-(4-carboxymethylphenyl) porphyrin, was evaluated *in silico* and *in vitro.* The cellular uptake of the investigated porphyrin and its ability to perform photodynamic therapy were investigated in terms of the viability, proliferation, and necrosis of human HaCaT keratinocytes and human Hs27 skin fibroblasts, in correlation with the predictions regarding diffusion through cell membranes, ADMET profile (absorption, distribution, metabolism, elimination, toxicity), and potential pharmacological mechanism. Molecular docking and 250 ns molecular dynamics simulations revealed that P5.2 has the potential to form a relatively stable complex with the carbonic anhydrase IX catalytic site, the lowest predicted free energy of binding (MM/PBSA) being −39.097 kcal/mol. The results of the in vitro study showed that P5.2 is incorporated within 24 h in the investigated cells, especially in HaCaT keratinocytes, indicating its photosensitizing ability. Nevertheless, P5.2 does not exert significant cytotoxicity in “dark” conditions. In turn, PDT induced a decrease in the number of metabolically active HaCaT keratinocytes within 24 h, accompanied by a 4-fold increase in lactate dehydrogenase release, indicating its ability to perform PDT in human skin cells. The experimental results suggest that the asymmetrical porphyrin is a promising candidate theranostics agent for skin disorders.

## 1. Introduction

In recent years, the development of new photosensitizers with tetrapyrrolic structures has created a multitude of therapeutic perspectives, especially in cancer theranostics [1,2,3,4,5,6]. Porphyrin-type derivatives show good affinity in relation to tumor cells, proving their potential use in oncology. Their structural design, photophysical profile, and strong absorption capacity in the therapeutic window, correlated with their ability to generate reactive oxygen species (ROS) in the presence of molecular oxygen and light, are the main factors behind their therapeutic potential [1,5,7,8,9].

Photodynamic therapy (PDT) with porphyrin-type derivatives is frequently used in dermatology to treat various skin disorders such as actinic keratosis (AK), in situ cutaneous squamous cell carcinomas (cSCCs), basal cell carcinomas (BCCs), cutaneous infections, inflammatory dermatoses, as well as cutaneous T-cell lymphoma [10,11,12].

PDT, as a non-invasive therapy with high selectivity for cancer cells and reduced side effects, is a promising alternative to conventional antitumor approaches (chemotherapy, radiotherapy). In PDT, the photosensitizer (PS) generates singlet oxygen following irradiation with visible light of a well-defined wavelength [7,8,9], leading to cell death.

Topical application of PSs offers the advantage of field PDT and decreases the risk of systemic phototoxicity [10,11].

Non-melanoma skin cancers (basal cell carcinoma and cutaneous squamous cell carcinoma) are among the most common types of cutaneous malignancies, and their incidence is continuously increasing [13,14,15,16,17,18,19].

Actinic keratosis (AK) is a precancerous form of cSCC, with a risk of malignancy up to 16% [20,21]. Actinic keratosis appears because of long-term exposure to ultraviolet radiation and the consequent proliferation of keratinocytes. Topical PDT using aminolevulinic acid (ALA) or its methyl ester is a frequently utilized treatment for AK and field cancerization [22]. However, the clinical effectiveness of ALA is limited due to its low cellular uptake and poor bioavailability, both attributed to its structural characteristics and hydrophilic nature [13].

In PDT-mediated keratolytic treatment, tetrapyrrole derivatives like Hematoporphyrin are used and have demonstrated superior efficacy to ALA [23]. Currently, various pharmaceutical products that incorporate PSs with tetrapyrrole structures (e.g., Radachlorin^®^, Purlytin^®^, Foslip^®^, Photofrin^®^) are utilized in topical PDT for cutaneous disorders [23,24].

The efficacy of photodynamic therapy (PDT) is based on factors such as light dosimetry, cellular internalization, duration of irradiation, and PS concentration in tumoral cells. Nevertheless, the internalization and distribution of PSs remain the most important parameters, with the epidermal penetration depth of PSs as the main indicator of drug efficacy in topical PDT [25].

Despite progress in the development of new PSs, PDT is currently limited by the poor tissue penetration of these structures. For increasing PS penetration into deep epidermal regions, innovative drug design strategies have been addressed. Proposals have been made concerning the chemical modification of PSs by adding polar and nonpolar functional groups in order to obtain an optimal hydrophilic/lipophilic ratio that can significantly increase their accumulation in diseased tissue.

Porphyrins have structural versatility and can be customized through the addition of substituents with various polarity degrees at tetrapyrrolic macrocycles and increasing the cellular uptake potential. Furthermore, in contrast to ALA, porphyrins do not need metabolization to become active PSs [26] and have a remarkable ability to generate singlet oxygen upon interaction with molecular oxygen and light [5,6,9,27,28,29]. In addition, the fluorescence properties of porphyrins, together with excellent tumor cell targeting ability, expand their applicability in the clinical diagnosis and imaging of malignant skin lesions [26,28,30].

Using ecological and versatile approaches, our research group obtained and characterized a panel of new asymmetrical porphyrins with a general profile suitable for theranostics applications [31,32,33,34,35,36,37,38].

**Figure 1:**
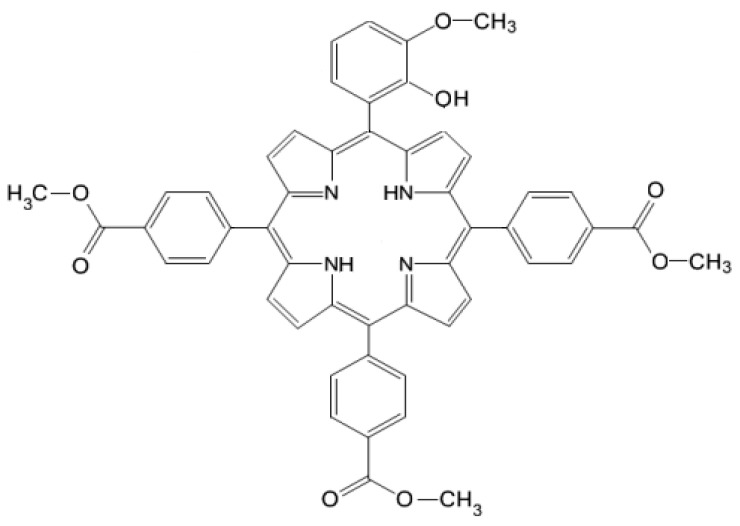
The molecular structure of the 5-(2-hydroxy-3-methoxyphenyl)-10,15,20-*tris*-(4-carboxymethylphenyl) porphyrin.

Considering the importance of developing new photosensitizers with suitable properties for PDT in non-malignant and malignant skin disorders, in this study, we performed an *in silico* and *in vitro* evaluation of an A_3_B-type porphyrin structure, namely 5-(2-hydroxy-3-methoxyphenyl)-10,15,20-*tris*-(4-carboxymethylphenyl) porphyrin (P5.2) (Figure 1).

The choice of this structural architecture was imposed by the need to ensure good cellular internalization of PS. The presence of functional groups (–OH, –OCOCH_3_, –OCH_3_) in the porphyrin structure increases the potential of the molecule to generate intermolecular hydrogen bonds, with increased solubility in polar environments. Furthermore, the –OH group improved the compound’s ability to dissolve in PEG 200, a frequently utilized solvent in pharmaceutical formulations. The reason for selecting an A_3_B-type structure was the necessity to achieve a compromise between solubility in biological environments and maintaining the relevant spectral properties for applicability in PDT.

Computational studies were used to predict the interaction of P5.2 with biological membranes and determine its ADMET profile. P5.2 was subjected to additional investigation using human skin-relevant cell lines, including HaCaT keratinocytes and Hs27 fibroblasts, to assess the cellular uptake of P5.2 and its ability to perform PDT in vitro

## 2. Results and Discussion

### 2.1. Computational Studies

#### 2.1.1. Prediction of Diffusion through the Cell Membrane

**Figure 2:**
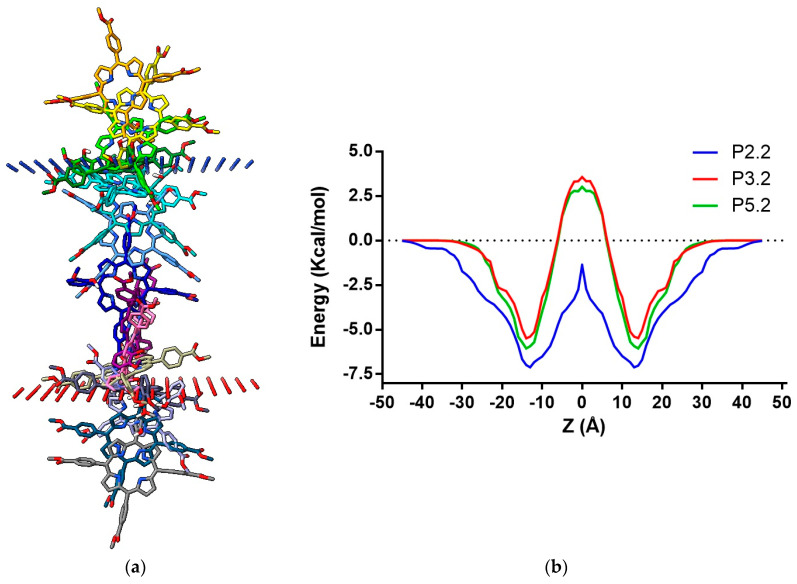
(**a**) Predicted translocation pathway across the lipid bilayer for P5.2; (**b**) variation in the transfer energy (ΔG_transf_) as a function of distance from the center of the lipid bilayer (Z) for P5.2 in comparison with P2.2 and P3.2.

The ability of the porphyrin compound P5.2 to cross the cell membrane was predicted using the PerMM (Permeability of Molecules across Membranes) web server, by simulating the translocation of the P5.2 structure through the lipid bilayer consisting of dioleoyl phosphatidylcholine. The used software allowed the calculation of the binding energy of P5.2 to the cell membrane (ΔG, kcal/mol) structure, the permeability coefficient (logPerm), and the generation of the transfer energy (ΔG_transf_) profile as a function of the distance from the center of the membrane (Figure 2). The prediction results were compared with the transfer energy profiles of two other asymmetrical porphyrin derivatives, 5-(4-hydroxy-3-methoxyphenyl)-10,15,20-*tris*-(4-acetoxy-3-methoxyphenyl) porphyrin (P2.2) and 5-(2-hydroxy-5-methoxyphenyl)-10,15,20-*tris*-(4-carboxymethylphenyl) porphyrin (P3.2), examined in our previous work [31].

The simulation results indicated a predicted value of the cell membrane binding energy (ΔG = −6.06 kcal/mol) and permeability coefficient (logPerm = −2.7), suggesting relatively good membrane permeability (Figure 2). The transfer energy profile of P5.2 was more comparable to that of P3.2, requiring similar energies to diffuse through the water molecules (±15–30 Å from the bilayer center) and to cross the hydrophobic core of the membrane (15 Å from the bilayer center). The binding energy of P5.2 was slightly lower than the energy of P3.2 (−5.49 kcal/mol) and higher than P2.2 (−7.15 kcal/mol). Moreover, the predicted permeability coefficient of P5.2 was lower than the estimated value of P2.2 (1.28) but higher than the permeability coefficient of P3.2 (−3.08). Therefore, P5.2 should cross the cell membrane in a similar manner to P3.2, but with greater difficulty than P2.2.

#### 2.1.2. Prediction of ADMET Profile for Porphyrin Derivative P5.2

A preliminary computational evaluation of the ADMET profile (absorption, distribution, metabolism, elimination, toxicity) was performed, estimating several pharmacokinetic and toxicological parameters for the porphyrin derivative P5.2. Predictive models used to estimate the pharmacokinetic properties of the tetrapyrrolic compound showed good intestinal absorption potential and increased oral bioavailability (Table 1).

In addition, the models used indicated a good potential for diffusion through the skin, which is relevant in the context of managing premalignant or malignant skin lesions through PDT. After diffusion through the cell membrane, the compound showed the potential for localization at the mitochondrion, thus being able to exert its cytotoxic effect by producing mitochondrial dysfunctions. Predictions also highlighted the inhibitory potential of P5.2 on cytochrome P450 isoforms. The compound can also bind to plasma proteins (approximately 88%) and is highly likely to cross the blood–brain barrier. For the P5.2 compound, a high logarithmic value of total clearance was predicted.

The predicted LD50 value was equal to 3066 mg/kg (Table 2), placing P5.2 in toxicity class V (potentially toxic after systemic administration). Class I toxicity represents the most toxic, lethal compounds after ingestion and class VI is specific to non-toxic compounds with LD50 > 5000 mg/kg. The predicted maximum tolerated dose is 2.74 mg/kg. The investigated porphyrin derivative has a very low probability of producing skin sensitization after topical administration, and this aspect is relevant for the applicability of the porphyrin compound, namely the PDT of premalignant and malignant skin disorders. On the other hand, the predictive models indicated potential hepatotoxicity, nephrotoxicity, mitochondrial toxicity, reproductive toxicity and immunotoxicity, but with relatively low probabilities, except for immunotoxicity. Considering that the potential immunotoxicity of the porphyrin compound could be detrimental in the context of oncologic patients, further studies are warranted to probe the prediction results.

#### 2.1.3. Molecular Docking

The potential antitumor activity of P5.2 was evaluated by simulating the interaction with carbonic anhydrase IX (CAIX), a cell-surface glycoprotein that is a biological target involved in acidification of the extracellular matrix, tumorigenesis, and metastasis, and its activity is induced by hypoxia. CAIX is a zinc metalloenzyme that catalyzes the interconversion between carbon dioxide, bicarbonate anions, and protons and is overexpressed in malignant cells, being proposed as a promising target for the treatment of several types of malignancies, including non-melanoma skin cancers [39,40].

The active site of CAIX is formed by the catalytic Zn^2+^, which is complexed with three histidine residues (His94, His96, His119), the polar residues Glu106, Asp131, Thr200, Thr201, and Gln92, and multiple hydrophobic residues that form the binding pocket, such as Val121, Val130, Leu199, Pro203, and Trp210.

**Figure 3:**
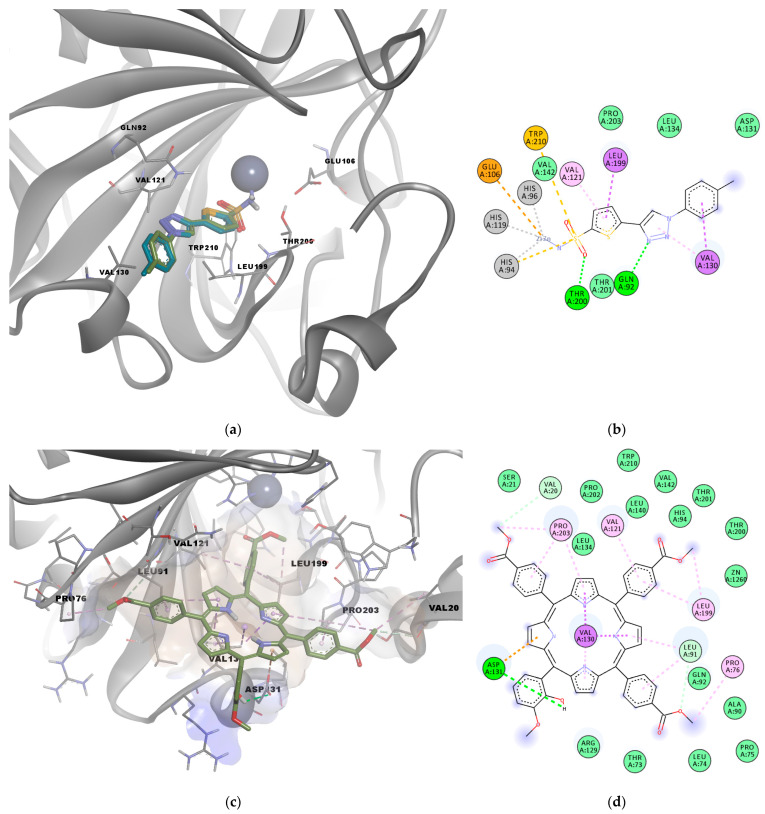
(**a**) Superposition of predicted binding pose of the co-crystallized sulfonamide CAIX inhibitor (Y0R, blue) on the original experimental structure (green); (**b**) predicted molecular interactions for the redocked sulfonamide CAIX inhibitor; (**c**) 3D conformation of the predicted complex between P5.2 and CAIX; (**d**) 2D diagram of predicted interactions between P5.2 and CAIX. Green dashes—hydrogen bonds; grey dashes—metal–acceptor bonds; tea green dashes—carbon–hydrogen bonds; orange dashes—pi–anion interactions; purple dashes—pi–sigma interactions; pink dashes—alkyl or pi–alkyl interactions; yellow dashes—pi–sulfur interactions; light green circles—van der Waals interactions.

The docking protocol was first validated by simulating the interaction between the co-crystallized sulfonamide inhibitor Y0R (positive control) and CAIX. The known inhibitor had a binding energy of −7.935 kcal/mol. The docked pose of the positive control molecule had a deviation from the original structure of only 0.5169 Å, showing good accuracy of the algorithm in predicting the correct binding pose (Figure 3a). The predicted molecular interactions were also similar to those observed in the experimental structure; since a coordinate bond was formed with the catalytic zinc, hydrogen bonds were formed with Thr200 and Gln92, a pi–sulfur interaction with Trp210, and other hydrophobic interactions with Val121, Val130, and Leu199 (Figure 3b).

The molecular docking experiment performed for the investigated porphyrin yielded a binding energy of −9.046 kcal/mol in the active site of CAIX. Analysis of predicted interactions between P5.2 and CAIX suggests the existence of physical hydrogen bonds with Asp131 and carbon–hydrogen bonds with Leu91 and Val20 that stabilize the P5.2-CAIX association. In addition, the data obtained indicate the formation of an electrostatic interaction (pi–anion) between Asp131 and a pyrrolic nucleus. The other three pyrrolic nuclei of the porphyrin structure showed the potential to interact via hydrophobic interactions with Val130, such as pi–sigma and pi–alkyl interactions. Moreover, several other hydrophobic contacts were revealed between the porphyrin derivative and residues Pro76, Leu199, Val121, Pro203 (alkyl or pi–alkyl interactions), and other amino acids within the binding pocket (van der Waals interactions, Figure 3a,b). One carboxymethyl moiety was also in close contact with His94 and the catalytic Zn^2+^.

#### 2.1.4. Molecular Dynamics Simulations

**Figure 4:**
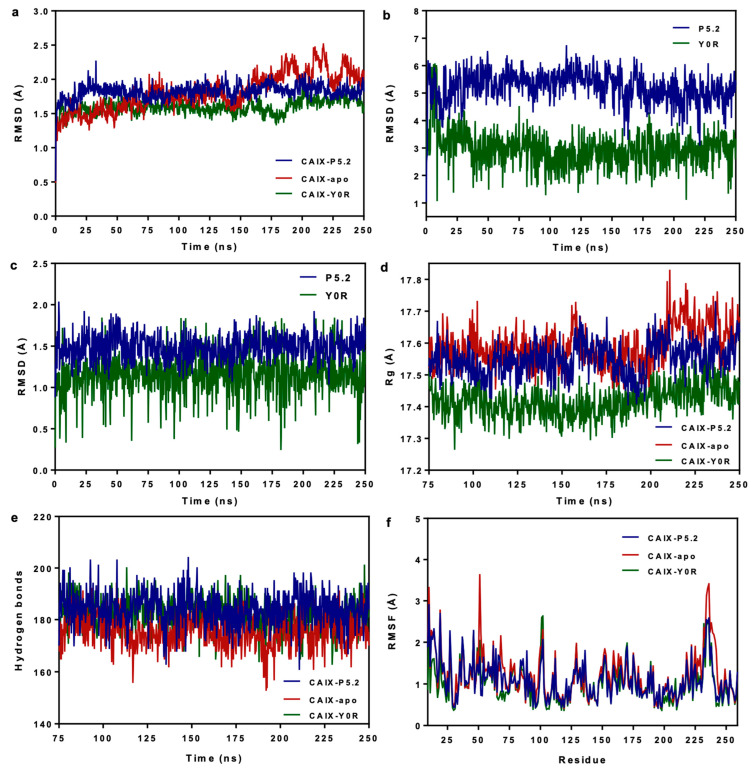
MD results after 250 ns of simulation time. (**a**) RMSD variation of all protein carbon atoms in relation to simulation time for CAIX-P5.2 vs. positive and negative controls; (**b**) ligand movement RMSD after superposing on the receptor, illustrating the movement of P5.2 and Y0R in the binding pocket; (**c**) ligand conformation RMSD after superposing on the initial coordinates, illustrating the conformational changes of P5.2 and Y0R; (**d**) radius of gyration (Rg) for CAIX-P5.2 vs. positive and negative controls; (**e**) number of intramolecular hydrogen bonds for the CAIX-P5.2 complex vs. positive and negative controls; (**f**) RMSF values per amino acid residue for the CAIX-P5.2 complex vs. positive and negative controls.

The predicted protein–ligand complex between CAIX and the tetrapyrrolic compound P5.2 was further analyzed by assessing the stability over time through molecular dynamics (MD) simulations. The predictions were carried out for 250 ns and the results were compared with the trajectories of the apo structure of CAIX (negative control) and the CAIX complex with the sulfonamide inhibitor Y0R (positive control). MD simulations revealed that the CAIX-P5.2 was more stable over time than the apo protein, since the root mean square deviation (RMSD) of protein carbon atoms had lower variations. Moreover, both protein–ligand complexes reached a relative equilibrium at 25 ns, while the equilibration phase of the ligand-free structure was considered from 0 to 75 ns. (Figure 4a). The CAIX–porphyrin complex showed similar, relatively stable behavior to the complex with Y0R, although the atom deviations were higher. Considering the slower equilibration of the apo protein, we performed further analysis on the last 175 ns of simulation time, which was considered the production phase. Ligand conformation and ligand movement RMSD variations were relatively stable after the equilibration period (Figure 4b,c), with the positive control inhibitor showing lower values, possibly due to the metal coordinate bond and the considerably smaller structure. Interestingly, the radius of gyration (Rg) values were on average lower for the CAIX-P5.2 and CAIX–sulfonamide complexes than the radii of the apo structure (Figure 4d), while higher numbers of intramolecular hydrogen bonds were noted for the protein–ligand complexes (Figure 4e), suggesting that the ligand-bound protein structures adopted relatively more compact conformations during the simulation time. Root mean square fluctuation values corresponding to each amino acid residue are shown in Figure 4f. Moreover, relatively lower RMSF values were observed for relevant residues that were involved in interactions with P5.2 or the known inhibitor when compared to the values calculated for the free protein structure. For instance, RMSF values of 1.28 Å (Pro76), 1.02 Å (Val130), and 1.15 Å (Asp131) were recorded for the CAIX-P5.2 complex, compared to 1.59 Å (Pro76), 1.27 Å (Val130), and 1.32 Å (Asp131) for the control.

**Figure 5:**
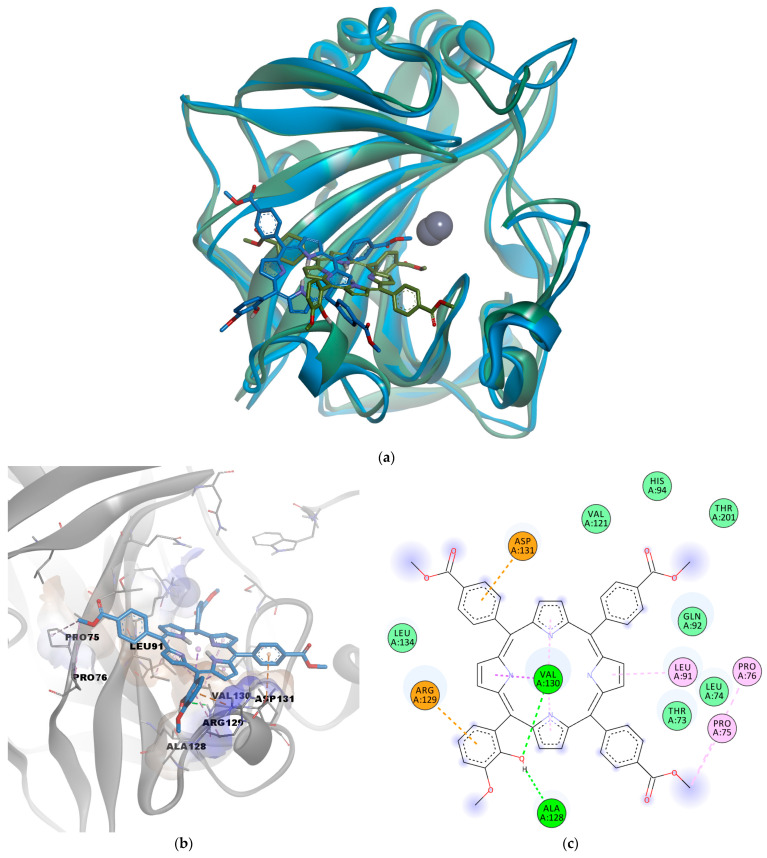
(**a**) Superposition of simulated CAIX-P5.2 complex (blue) with lowest ligand binding energy on initial conformation (green); (**b**) binding pose of P5.2 corresponding to lowest energy snapshot; (**c**) 2D diagram of predicted molecular interactions between CAIX and P5.2 corresponding to the lowest binding energy snapshot. Green dashes—hydrogen bonds; orange dashes—pi–anion/pi–cation interactions; pink dashes—alkyl or pi–alkyl interactions; light green circles—van der Waals interactions.

The predicted binding pose of P5.2 with the lowest free energy of binding was further discussed. The MM/PPBSA free energy estimation approach revealed a binding energy of –39.097 kcal/mol for the selected conformation. The superposition of the corresponding conformation of the simulated complex on the starting conformation is depicted in Figure 5a, while the interactions with the binding site residues are shown in Figure 5b,c. Notably, the hydrogen bond between the phenolic hydroxyl of P5.2 and Asp131 was lost during the simulation, but the same moiety engaged in two other hydrogen bonds with Ala128 and Val 130. However, Asp131 formed a pi–anion electrostatic interaction with a phenyl ring, instead of the pyrrole substructure. Moreover, Arg129 formed a pi–cation interaction with the hydroxylated phenyl radical, while Val130 formed hydrophobic pi–sigma and pi–alkyl interactions with the tetrapyrrolic ring, similar to the initial conformation. A previous study also highlighted the potential interactions between another porphyrin derivative (5,10,15-*tris*(tolyl)-20-mono(p-nitrophenyl) porphyrin, TrTMNP) and CAIX, which also engaged in pi–anion interactions with Asp131 [41]. The simulation data suggest that the porphyrin derivative P5.2 has the potential to inhibit the catalytic activity of CAIX by forming a relatively stable complex with the active site, even if no direct coordinate bonds are formed with the catalytic zinc. The predicted inhibitory activity on the overexpressed CAIX could potentially act synergistically with in vivo PDT to induce apoptosis in non-melanoma skin cancer cells.

### 2.2. Photophysical Characterization of Porphyrinic Compound

**Figure 6:**
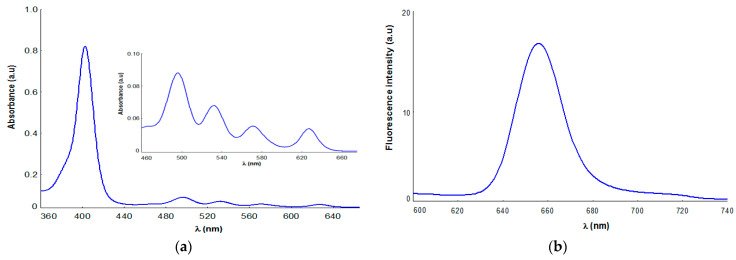
(**a**) Absorption spectrum of P5.2 dissolved in PEG 200, c = 10 µM. The insets show magnification of the corresponding Q band region. (**b**) Fluorescence spectrum of P5.2 at a concentration of 10 µM in PEG 200 as a solvent; λex = 410 nm.

Taking into account the fact that the absorption and emission properties of porphyrin compounds are the most relevant from the point of view of their applicability in photodynamic therapy, in this study, we present some data associated with the UV-Vis and fluorescence profile of P52, registered for 10µM P5.2 porphyrin dissolved in polyethylene glycol 200. Thus, the molecular absorption spectra of P5.2 (Figure 6a) exhibit the typical spectral features of free-base porphyrins [42,43,44], with an intense Soret band at 402 nm, accompanied by other four Q bands in the 496–630 nm spectral region.

The presence of a Q band at 630 nm in the UV-Vis spectrum of P5.2 (Figure 6a) confirms its absorption potential in the phototherapeutic field, a spectral range of interest for PDT applications [9,29].

Fluorescence emission spectra (Figure 6b) were recorded in PEG 200 as a solvent at a concentration of 10 µM P5.2 and displayed an emission maximum at 657 nm. It should be noted that P52 emission properties are associated with a typical spectrum porphyrinic photosensitizer, with good fluorescence properties and a promising theranostics potential in cutaneous disorders [9,28,29,30].

### 2.3. In Vitro Studies

A preliminary study on the biological activity of P5.2 was performed using the human skin cell lines HaCaT keratinocytes and Hs27 fibroblasts. P5.2 cell uptake; MTS reduction, informing on the number of metabolically active cells in culture; and the lactate dehydrogenase (LDH) release, informing on cell death, were investigated.

#### 2.3.1. P5.2. Uptake in Human Keratinocytes and Fibroblasts

**Figure 7:**
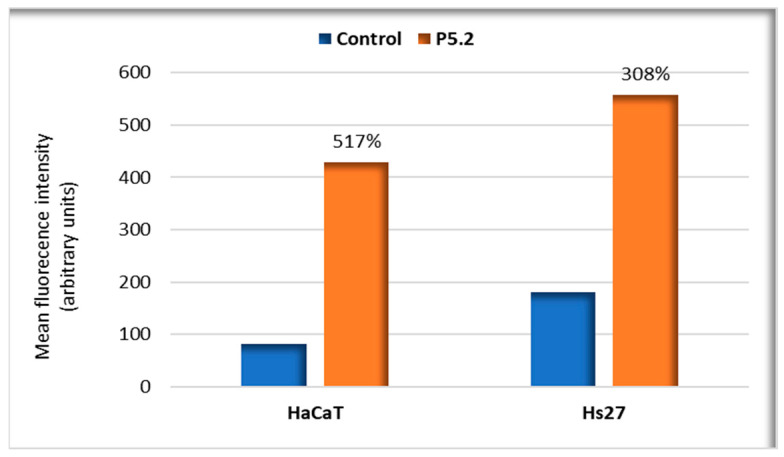
Uptake of P5.2 (10 µM) by human skin cells (HaCaT keratinocytes and Hs27 fibroblasts). The uptake was evaluated by flow cytometry after 24 h incubation with P5.2. The results are expressed as mean fluorescence intensity in the FL3 channel for a demonstrative experiment. The percentage of the mean fluorescence intensity in P5.2-treated cells relative to the control is provided.

Flow cytometry data showed that the fluorescent P5.2 was incorporated in 24 h in the investigated cell lines, especially in HaCaT keratinocytes, as seen by an increase in cellular fluorescence of 517% in HaCaT keratinocytes and of 308% in Hs27 fibroblasts (Figure 7). The results confirm the potential utility of the porphyrin derivative as a fluorescent marker in normal or malignant skin cells. Moreover, the cellular uptake of the porphyrin derivative is also an essential condition for its potential use as a PS in PDT.

#### 2.3.2. Biocompatibility in Human Keratinocytes and Fibroblasts

**Figure 8:**
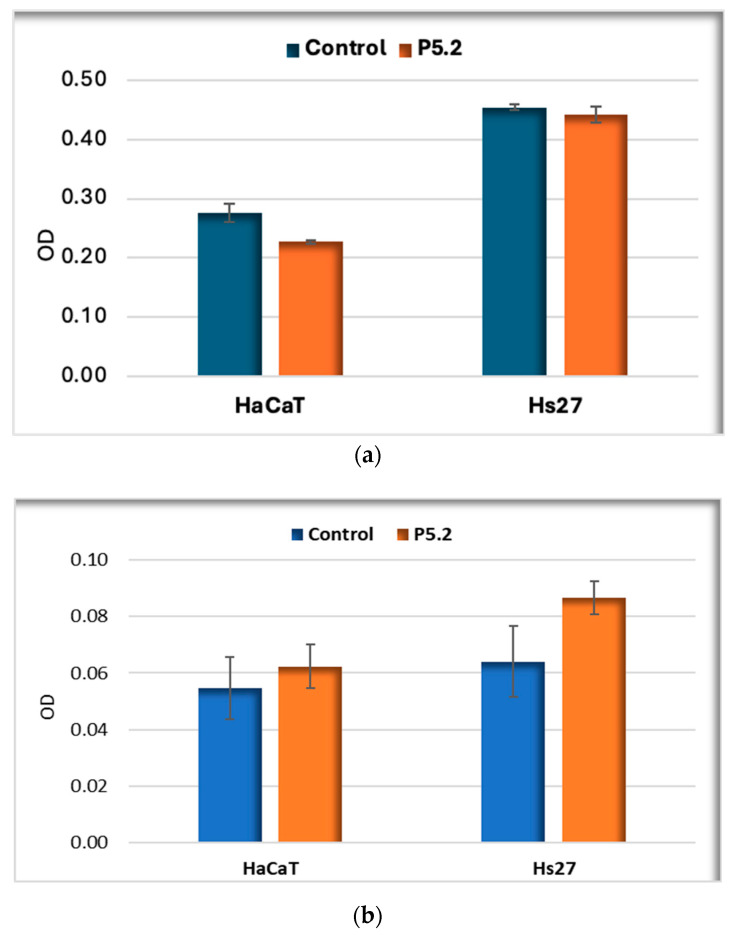
MTS reduction (**a**) and LDH release (**b**) by normal human skin cell lines (HaCaT keratinocytes and Hs27 fibroblasts) treated for 24 h with 10 µM P5.2. The results are presented as mean OD value ± SEM for triplicate samples.

P5.2 was not cytotoxic in “dark” conditions against human skin cells, as evidenced by the lack of statistical changes in MTS reduction (Figure 8a) and LDH release (Figure 8b). However, there was a more pronounced tendency to release LDH in the case of Hs27 human skin fibroblasts compared to HaCaT keratinocytes (Figure 8b).

The effect exerted in vitro by P5.2 on the number of active metabolic cells was also evaluated as a measure of its potential anti-proliferative effects. MTS reduction data showed nominally lower MTS reductions by normal human keratinocytes (*p* < 0.05), while human dermal fibroblasts were not affected at the level of this parameter.

#### 2.3.3. Photodynamic Therapy in Human Keratinocytes

**Figure 9:**
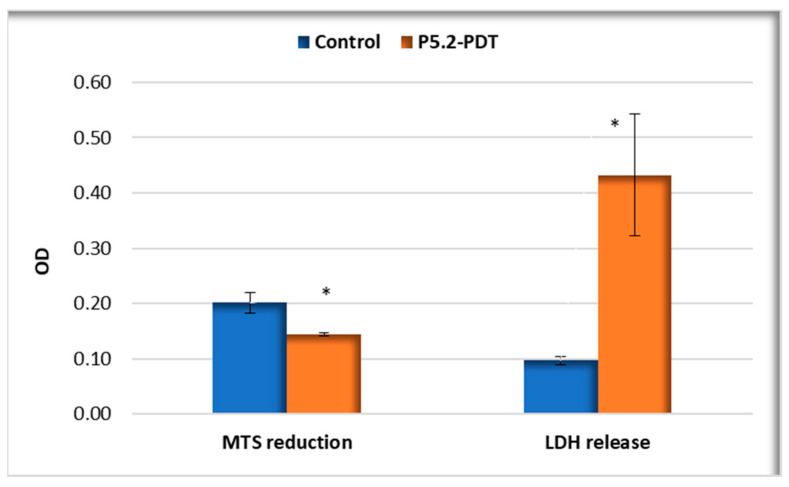
MTS reduction and LDH release by HaCaT keratinocytes loaded with P5.2 (10 µM), subjected to PDT (10 J/cm^2^, 50 mW/cm^2^) and further analyzed at 24 h after treatment. The results are presented as mean OD value ± SEM for triplicate samples. * *p* < 0.05 (Student *t*-test).

The ability of P5.2 to perform PDT in vitro when activated with 635 nm light was investigated on HaCaT keratinocytes that were shown to incorporate P2.2 better than Hs27 fibroblasts (Figure 7). MTS reduction data showed a decrease to 72% of the number of metabolically active cells at 24 h after PDT, accompanied by an approximately 3-fold increase in LDH release (Figure 9). Therefore, experimental data highlight the potential of P5.2 to induce a moderate photosensitization of normal keratinocytes. Further studies are warranted to investigate the cytotoxic activity on malignant keratinocytes.

## 3. Materials and Methods

### 3.1. General Information

Commercially available chemicals and solvents from Sigma-Aldrich (St. Louis, MO, USA) and Merck (Whitehouse Station, NJ, USA) were used. The P5.2 asymmetrical porphyrin was synthesized according to the method proposed by the authors [45]. The compounds used as references, P2.2 and P3.2, were also reported elsewhere [31,32].

The spectral profile of P5.2 was evaluated through UV–Visible and fluorescence spectroscopy techniques. UV-Vis spectra were obtained using a Specord 200 spectrophotometer (Analytik Jena, Jena, Germany). Fluorescence spectra were obtained using a Jasco FP 6500 spectrofluorometer (JASCO Co., Ltd., Kyoto, Japan), in 10 mm path-length quartz cuvettes.

### 3.2. Computational Studies

#### 3.2.1. Permeability through Cell Membrane Prediction

Membrane permeability in relation to the structure of P5.2 was predicted with the PerMM (Permeability of Molecules across Membranes) web server (University of Michigan, Ann Arbor, MI, USA) [46]. The three-dimensional structure of the porphyrin derivative was generated using Data Warrior [47] and the lowest-energy conformation was generated by energy minimization with the MMFF94s+ force field. The simulation was executed at a temperature of 298 K and at pH = 7.4. The deionization energy was not taken into consideration, as the studied compound protonate in acidic medium at pH lower than 6. The binding energy (ΔG, kcal/mol) and permeability coefficient (logPerm) were calculated, and the transfer energy profile was generated as a function of the distance from the membrane center. The results were compared with two other porphyrin derivatives that were investigated in our previous study, P2.2 and P3.2 [31].

#### 3.2.2. ADMET Profile Prediction

The ADMET (absorption, distribution, metabolism, excretion, and toxicity) profile of the porphyrin derivative was predicted using three different web servers: pkCSM, admeSAR, and ProTox-II [48,49,50]. Both local and systemic administrations were taken into consideration.

#### 3.2.3. Molecular Docking Studies

Molecular docking simulations were carried out to predict the inhibitory potential of P5.2 on carbonic anhydrase IX (CAIX). The crystal structures of CAIX (PDB code 5FL6, 1.96 Å resolution) in complex with a sulfonamide inhibitor (Y0R, 5-[1-(4-methylphenyl)-1,2,3-triazol-4-yl]thiophene-2-sulfonamide) was retrieved from the RCSB PDB database [51,52]. Protein preparation steps and molecular docking studies were carried out using the YASARA Structure software [53]. The protein structure was processed for docking by removing solvent and ligand molecules, protonation according to the physiological pH (7,4), optimization of the hydrogen-bond network, and energy minimization using the YASARA2 force field. 

The evaluated porphyrin derivative was prepared by generation of the 3D structure and energy minimization with the MMFF94s+ force field, using DataWarrior. The docking calculations were performed in a blind manner using the AutoDock Vina v1.1.2 algorithm [54] with flexible sidechains. The grid box (70 × 70 × 70 Å) was placed to include the whole protein structure. The co-crystallized sulfonamide CAIX inhibitor (Y0R) was also docked into the binding site to validate the docking protocol, and the predicted binding mode was superposed on the original structure to calculate the root mean square deviation (RMSD). The results were expressed as binding energy (ΔG, kcal/mol) and the predicted molecular interactions between the protein and ligand were evaluated using the BIOVIA Discovery Studio Visualizer 17.2.0 software (BIOVIA, Discovery Studio Visualizer, Version 17.2.0, Dassault Systèmes, 2016, San Diego, CA, USA).

#### 3.2.4. Molecular Dynamics Simulations

Molecular dynamics (MD) simulations were carried out to evaluate the stability of the P5.2-CAIX complex obtained via molecular docking. The unbound (apo) structure of CAIX was also simulated as a negative control structure, while the docked sulfonamide inhibitor was used as a positive control structure. Simulations were performed using YASARA Structure, for a total duration of 250 ns, similar to our previous studies [55,56]. The system stability was increased by optimizing the hydrogen bonding network. Simulation cells were neutralized by adding 0.9% NaCl, and steepest descent and simulated annealing minimizations were performed to remove clashes. AMBER14 force field was used for the protein, GAFF2 and AM1BCC for the ligand, and the TIP3P force field for water. The cut-off for van der Waals forces was set to 8 Å, and the electrostatic forces were treated with the Particle Mesh Ewald algorithm and no cut-off. The integration of motion equations was performed with a multiple timestep of 2.5 fs for bonded and 5 fs for non-bonded interactions, at 298 K and 1 atm (isothermal-isobaric ensemble). The free energy of binding (kcal/mol) of the simulated porphyrin derivative was calculated using the Poisson–Boltzmann (MM/PBSA) approach, excluding the entropic term.

### 3.3. In Vitro Studies

#### 3.3.1. Cells

The human skin cell lines HaCaT keratinocytes (CLS Cell Lines Service GmbH, Eppelheim, Germany) and Hs27 fibroblasts (Hs27-CRL-1634, ATCC, Manassas, VA, USA) were used. Cells were maintained in culture in DMEM-F12 culture medium (Gibco, Thermo Fisher Scientific, Waltham, MA, USA), supplemented with 10% fetal bovine serum (FBS, Sigma, Saint Louis, MO, USA) and an antibiotic–antimycotic solution (Sigma, Burlington, MA, USA), further nominated as complete culture medium.

Porphyrinic compound. P5.2 was dissolved in PEG 200 at a concentration of 10 mM, and the solution was stored at room temperature in the dark. Before experiments, the stock solution was diluted to a final concentration of 10 µM in DMEM-F12 culture medium (Gibco, Thermo Fisher Scientific, Waltham, MA, USA), supplemented with 2% FBS (Sigma, Saint Louis, MO, USA) and an antibiotic–antimycotic solution (Sigma, Burlington, MA, USA).

#### 3.3.2. P5.2 Uptake

Due to the intrinsic fluorescence of P5.2, its uptake was evaluated by flow cytometry as follows. Cells (5 × 10^4^ HaCaT keratinocytes and 10 × 10^4^ Hs27 fibroblasts) were cultivated in complete culture medium in 24-well plates for 24 h at 37 °C in a 5% CO_2_ atmosphere to allow cells to adhere. The supernatant was then discarded, and a solution of 10 µM P5.2, prepared as described above, was added. The control samples did not contain P5.2. Cells were cultivated for another 24 h to allow for the cellular uptake of P5.2. Finally, the supernatant was discarded, and cells were detached with 0.25%/0.02% Trypsin/EDTA (Biochrom AG, Merck Millipore, Burlington, MA, USA) and suspended in Live Cell Imaging (Thermo Fisher Scientific, Waltham, MA, USA). Cells were analyzed by flow cytometry using a BD FACS Canto flow cytometer (Becton Dickinson, Franklin Lakes, NJ, USA) equipped with a 488 nm laser. The emission was registered in the FL3 channel (red). Flow cytometry data were acquired and processed with the BD FACSDiva software v6.1.3 (Waltham, MA, USA). The results were expressed as mean fluorescence intensity (arbitrary units) for 10,000 cells in each sample.

#### 3.3.3. Biocompatibility Assays

P5.2 biocompatibility was evaluated by the MTS [(3-(4,5-dimethylthiazol-2-yl)-5-(3-carboxymethoxyphenyl)-2-(4-sulfophenyl)-2H-tetrazolium)] reduction test, which provides information on the number of metabolically active cells in culture, complemented with the lactate dehydrogenase (LDH) release assay, which offers information about the alteration of the plasma membrane integrity in relation to necrotic cell death. The kits used were the CellTiter 96^®^ AQueous One Solution Cell Proliferation Assay and the CytoTox 96^®^ Non-Radioactive Cytotoxicity Assay from Promega Corporation (Madison, WI, USA), respectively. Cells were cultivated in triplicates in 96-well plates (5 × 10^3^ HaCaT keratinocytes and 10 × 10^3^ Hs27 fibroblasts in 100 µL complete culture medium) for 24 h at 37 °C in a 5% CO_2_ atmosphere. The supernatant was discarded, and a solution of 10 µM P5.2, prepared as described above, was added. Control samples, which did not contain P5.2, and background samples, which contained only complete culture medium, were prepared. The cultivation of samples continued for another 24 h. Plates were finally centrifuged for 5 min at 200 g at room temperature.

For the LDH release assay, 50 µL of supernatant was collected from each sample and transferred to a 96-well plate. A 50 µL volume of LDH substrate was added to the supernatants, and samples were incubated for 30 min at room temperature in the dark. The reaction was stopped with the solution contained in the kit. The colorimetric reaction was measured as described below.

For the MTS reduction test, 50 µL complete culture medium was added to the original culture plate to restore the 100 µL total volume of the culture. In each sample, 20 µL MTS was added, and cells were incubated for 90 min at 37 °C in a 5% CO_2_ atmosphere. The colorimetric reaction was measured as described below.

Colorimetric reactions were measured using a Tecan ELISA reader (Männedorf, Switzerland) at 490 nm against a 620 nm reference wavelength for the MTS reduction test, and at 490 nm for the LDH release test. Optical density (OD) data were acquired and processed using Magellan software v6.0 (Tecan, Männedorf, Switzerland). The OD values in experimental and control samples were corrected by subtracting the mean OD of background samples. The results were presented as mean corrected OD ± standard error of the mean (SEM) for triplicate samples.

#### 3.3.4. Photodynamic Therapy

HaCaT keratinocytes (0.5 × 10^6^ cells/mL) were cultivated for 24 h at 37 °C in a 5% CO_2_ atmosphere in Petri dishes with a diameter of 35 mm. Cells were then loaded for 24 h with 10 µM P5.2, prepared as described above. The supernatant was discarded and replaced with 1 mL HBSS supplemented with FBS and an antibiotic–antimycotic solution. Cells that were not exposed to PDT were considered as controls.

Photodynamic therapy (PDT) was applied to cell cultures using the Modulight ML6600 equipment (Modulight Inc, Tampere, Finland), equipped with a 635 nm laser, controlled in terms of fluence (10 J/cm^2^) and irradiance (50 mW/cm^2^) [57].

To evaluate the impact of PDT on cell viability, the exposure culture medium was discarded and replaced with complete culture medium, and cells were incubated for 24 h at 37 °C in a 5% CO_2_ atmosphere. Finally, cell-free supernatant was harvested from each sample for the LDH release assay. Cells were detached using 0.25%/0.02% Trypsin/EDTA (Biochrom AG, Merck Millipore, Burlington, MA, USA) and counted. For the MTS reduction test, the volume of cell suspension containing 20 × 10^3^ HaCaT cells in control samples was calculated. The same volume of cell suspension from experimental samples was harvested, irrespective of the cell number. The volume was adjusted to 100 µL with complete culture medium, and the MTS reduction test was performed as described above.

Data processing. Data were presented as mean value ± standard error of the mean (SEM) for triplicate samples. Comparisons between samples were performed using Student’s *t*-test considering unequal variances.

## 4. Conclusions

The porphyrin derivative P5.2 showed good biocompatibility with skin cells and the ability to perform PDT *in vitro*, making it a candidate for further development as a skin label and photosensitizer for dermato-oncologic applications in actinic keratosis. Molecular docking and molecular dynamics simulations supported the potential antitumor activity of P5.2, which could target the hypoxia-induced tumor-associated carbonic anhydrase IX. Further studies are needed to validate the in silico results and to investigate the cytotoxic activity of P5.2 in skin cancer cell lines.

## 5. Patents

a. Patent application 202200775: Andreea Mihaela Burloiu, Gina Manda, Rica Boscencu, Ionela Victoria Neagoe, Dumitru Lupuliasa, Mihaela Surcel, Laura Olariu, Mihai Dragos Paul, *Porphyrinic compound with fluorescent marker potential in dermato-oncology,* published in RO-BOPI, 5 from 30 May 2023.

## Figures and Tables

**Table 1:** Predicted ADME parameters for P5.2.

ADME Parameters	Activity Class/Predicted Value
Intestinal absorption	yes
P-glycoprotein P substrate	no
P-glycoprotein P inhibitor	yes
Oral bioavailability	high
Skin permeability	yes
Subcellular localization	mitochondria
CYP isoform inhibitor	yes (89.52%)
Plasma protein binding	88.1%
Blood–brain barrier permeability	yes
Total clearance	0.862 (log mL/min/kg)

**Table 2:** Predicted toxicity of P5.2.

Property	Class	Probability
Skin sensitization	inactive	0.9414
Hepatotoxicity	active	0.5250
Nephrotoxicity	active	0.6143
Carcinogenicity	inactive	0.5100
Mutagenicity	inactive	0.5000
Mitochondrial toxicity	active	0.6375
Reproductive toxicity	active	0.6111
Immunotoxicity	active	0.8800
Cytotoxicity	inactive	0.6300
LD50	3066 mg/kg (class V)	
Maximum tolerated dose	0.438 (log mg/kg/day)	

## Data Availability

Data are contained within the article.
